# Clinicians' Stress Levels During Soft Tissue Augmentation Using Autogenous Connective Tissue Grafts: A Pilot Study

**DOI:** 10.1111/cid.70130

**Published:** 2026-02-26

**Authors:** Anina N. Zuercher, Franz J. Strauss, Rawen Smirani, Ronald E. Jung, Reinhard Gruber, Daniel S. Thoma

**Affiliations:** ^1^ Clinic of Reconstructive Dentistry, Center for Dental Medicine, University of Zurich Zurich Switzerland; ^2^ Universidad Autonoma de Chile Santiago Chile; ^3^ University of Bordeaux, INSERM U1026 (BioTis), CHU Bordeaux, Université de Bordeaux Collège Sciences de la Santé Bordeaux France; ^4^ Department of Oral Biology University Clinic of Dentistry, Medical University of Vienna Vienna Austria; ^5^ Department of Periodontology Research Institute for Periodontal Regeneration, Yonsei University College of Dentistry Seoul Republic of Korea

**Keywords:** connective tissue graft, cortisol, heart rate, psychological questionnaire, soft tissue augmentation, stress

## Abstract

**Objectives:**

To quantify intraoperative stress responses in clinicians performing mucogingival surgery using subepithelial connective tissue grafts (SCTG) for mucosal thickening by combining physiological and psychological measures and to explore the feasibility of stress monitoring in this surgical context.

**Materials and Methods:**

Eleven clinicians performed 14 SCTG procedures. Physiological stress was assessed using heart rate (HR) and salivary cortisol measured at multiple time points from morning baseline through the evening. Psychological stress and workload were evaluated using validated questionnaires, namely the short‐form State–Trait Anxiety Inventory (STAI‐6) and the Surgery Task Load Index (SURG‐TLX). Data were analyzed with linear mixed‐effects models to account for repeated measures in this exploratory pilot study.

**Results:**

Heart rate showed a significant overall time effect (*p* < 0.001), with transient increases during incision, flap preparation and graft harvesting, followed by recovery during suturing. Salivary cortisol levels decreased progressively throughout the day (*p* < 0.01), consistent with normal diurnal variation and indicating the absence of sustained endocrine stress. Heart rate and cortisol patterns did not differ by clinician experience (< 3 years or ≥ 3 years). STAI‐6 scores remained stable over time, whereas SURG‐TLX responses identified high mental demand and task complexity as the dominant contributors to perceived workload.

**Conclusions:**

SCTG procedures are associated with short‐term physiological stress activation without concomitant increases in perceived anxiety, suggesting an adaptive stress and well‐regulated responses among clinicians.

**Clinical Significance:**

SCTG causes short‐term physical stress, but clinicians appear to cope well and do not feel more anxious while performing them. Characterizing stress dynamics during mucogingival surgery may help inform future research on clinician performance, fatigue, and procedural safety in soft tissue augmentation.

## 1 Introduction

Mucogingival surgery is a common procedure in periodontal and peri‐implant therapy, performed for root coverage, ridge augmentation and soft‐tissue enhancement around dental implants [1, 2, 3, 4]. These interventions aim to improve esthetics and maintain long‐term peri‐implant health [3, 5, 6]. The procedure typically involves flap preparation at the recipient site, harvesting of a subepithelial connective tissue graft (SCTG) from the palate and suturing of both surgical sites [5, 6, 7, 8].

Among these steps, flap elevation and graft harvesting are often perceived as the most technically and psychologically demanding. They require precise tissue handling, anatomical awareness and control under time constraints and suboptimal ergonomics, factors that may contribute to elevated intraoperative stress [9].

While intraoperative stress has been widely investigated in medicine [9, 10], little is known about its impact during mucogingival surgery on the clinicians' stress levels. Assessing clinician stress is clinically relevant, as stress may influence surgical performance, decision‐making and patient outcomes, although these relationships have not been established for SCTG procedures.

Stress can be evaluated using both physiological and psychological measures [9]. Physiological indicators include salivary cortisol and heart rate monitoring [11, 12], whereas validated questionnaires provide insights into perceived psychological stress [13]. Combining these approaches allows a more comprehensive evaluation of clinicians' intraoperative stress response. However, evidence on this topic remains scarce in periodontal and implant‐related surgery and no prior studies have focused specifically on SCTG procedures.

Therefore, this exploratory pilot study aimed to quantify intraoperative stress in clinicians performing mucogingival surgery with SCTG for soft‐tissue thickening. The primary goal was to describe stress patterns and evaluate the feasibility of monitoring stress during these procedures rather than to test cause‐and‐effect relationships.

## 2 Methods

Eleven clinicians, including postgraduate residents and senior faculty members from the Clinic of Reconstructive Dentistry at the University of Zurich, Switzerland, participated in this study. Demographic information such as age, gender and surgical experience was recorded. The Cantonal Ethics Committee of Zurich confirmed that no formal ethical approval was required for this project (BASEC Req. No. 2023‐01320).

**FIGURE 1:**
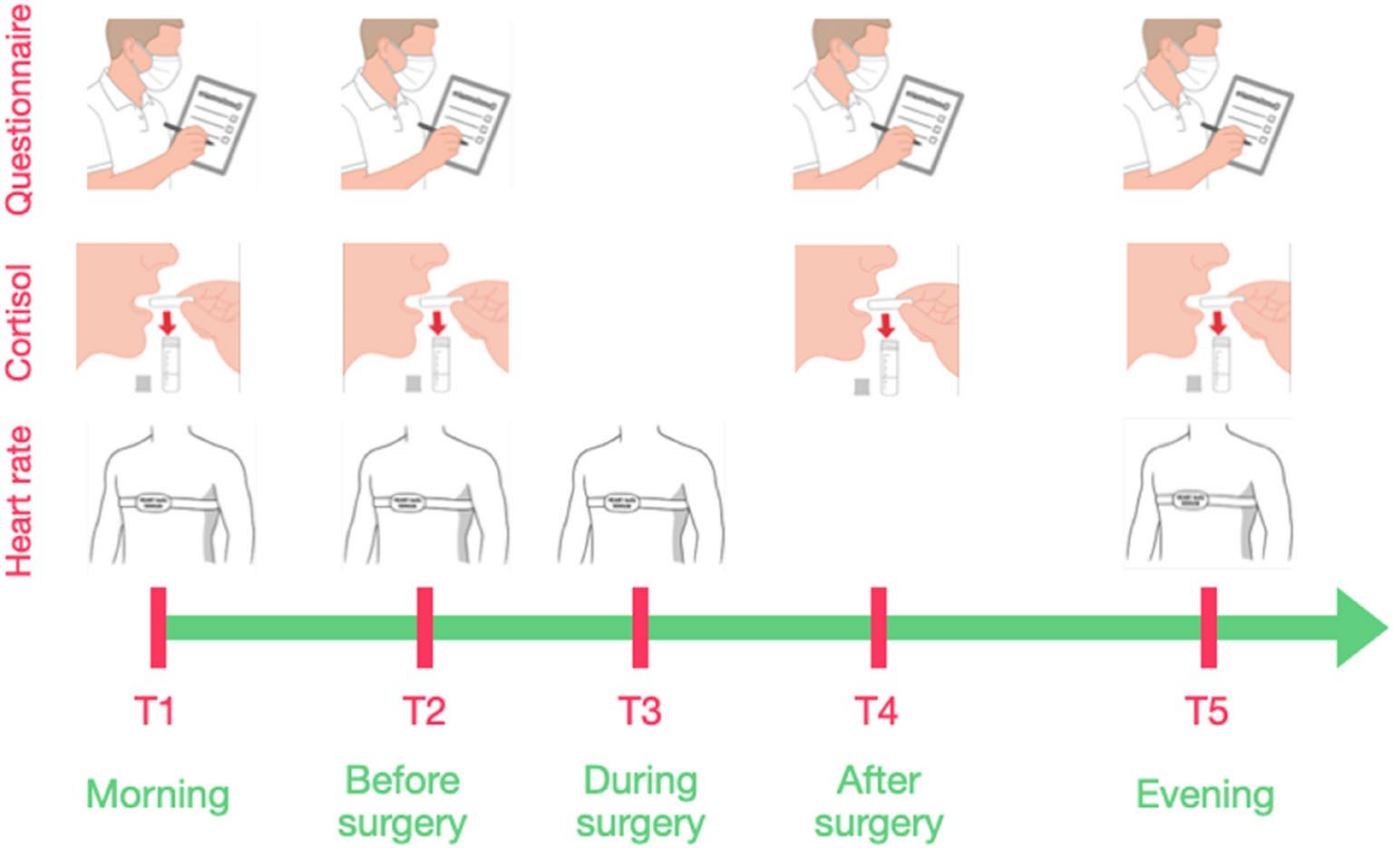
Timeline of data collection during soft tissue surgery. At morning baseline (T1), preoperative (T2), intraoperative (T3), postoperative (T4) and evening (T5). At each timepoint (T), stress was assessed using a self‐report questionnaire, salivary cortisol sample and continuous heart rate monitoring.

Intraoperative stress was assessed at five time points: T1 (morning baseline), T2 (10 min before surgery), T3 (during surgery), T4 (immediately after surgery) and T5 (evening of the procedure) (see Figure 1).

## 3 Outcome Measures

### 3.1 Physiological Measures

**FIGURE 2:**
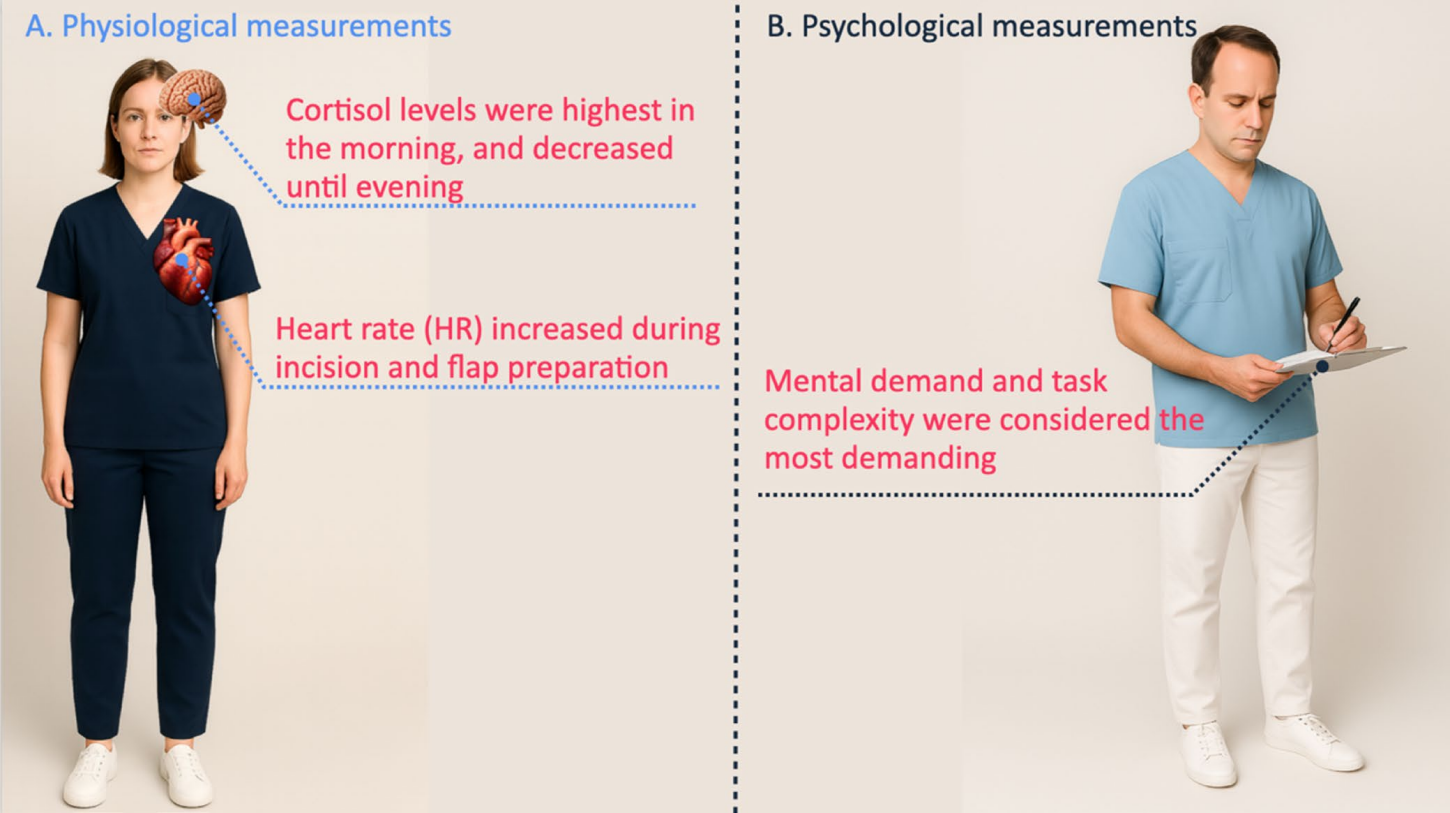
Physiological and psychological measures of clinicians during soft tissue surgery.

Physiological stress (Figure 2A) was evaluated using heart rate (HR) and salivary cortisol levels [11]. HR was recorded at T1, T2, T3 and T5 using a chest‐strap heart rate sensor (Polar H10, Polar Electro Oy, Kempele, Finland). Salivary cortisol samples were collected at T1, T2, T4 and T5 with sterile cotton swabs (Salivettes, Sarstedt, Germany) and processed according to standard laboratory protocols [14].

### 3.2 Psychological Measures

Psychological stress (Figure 2B) was assessed using two validated instruments. The short‐form *State–Trait Anxiety Inventory* (STAI‐6) was completed at T1, T2, T4 and T5 to evaluate transient anxiety levels [15]. The *Surgery Task Load Index* (SURG‐TLX) [13], adapted from the NASA‐TLX developed for pilots, was completed postoperatively to assess subjective workload.

### 3.3 Sample Size Calculation

Due to the exploratory nature of this study and the experimental setting, a formal sample size calculation was not performed. The study was intentionally designed as a pragmatic pilot to assess feasibility and to generate preliminary effect‐size estimates rather than to support confirmatory statistical testing. Accordingly, the sample size was chosen pragmatically based on clinician availability, with the aim of informing the design of future adequately powered studies.

### 3.4 Statistical Analysis

Descriptive statistics were calculated as means, standard deviations (SD) and medians for continuous variables and as frequencies for categorical variables. Changes in heart rate and cortisol levels over time were analyzed using linear mixed‐effects models to account for within‐clinician correlations of repeated measurements. Fixed factors included clinician experience (< 3 years or ≥ 3 years), time and their interaction, enabling estimation of effects at each time point, while individual clinicians were treated as random factors. Differences were assessed using linear contrast commands. These models were used to explore temporal patterns rather than to test confirmatory hypotheses. Model assumptions were evaluated visually through residual diagnostics, including Q–Q plots and histograms. The significance level (*α*) was set at 0.05 and all tests were two‐sided. Given the pilot nature of the study, *p*‐values are reported for completeness but should be interpreted with caution. Statistical analyses were performed using Stata version 18.0 (StataCorp, College Station, TX, USA).

## 4 Results

Eleven clinicians (four women, seven men) performed a total of 14 SCTG surgeries. Some clinicians contributed data from more than one surgery, resulting in repeated observations across time points. Heart rate measurements were not available for all procedures due to occasional technical issues, leading to sample sizes of *n* < 14 for HR‐related analyses (Tables 1, 2, 3). In contrast, salivary cortisol measurements were successfully completed for all procedures, and no missing data were observed for this outcome.

**TABLE 1:** Heart rate at each time point.

Variables	Timepoint	*n*	Mean (SD)	Median (Q1, Q3)
Heart rate (bpm)				
	T1	13	80.46 (11.31)	76.00 (73.00; 91.00)
	T2	13	87.62 (11.57)	87.00 (77.50; 96.50)
	T3	12	92.33 (10.29)	94.50 (83.75; 99.00)
	T5	9	73.44 (10.39)	72.00 (63.50; 84.00)

Abbreviations: nmol/L, nanomoles per liter; T, time points (morning baseline (T1), preoperative (T2), intraoperative (T3) and evening (T5)); SD, standard deviation; Q1, Q3, quartile 1, 3.

**TABLE 2:** Heart rate at each time point stratified by clinical experience.

Variables	Timepoint	*n*	< 3 years of experience	Median (Q1, Q3)	*n*	≥ 3 years of experience	Median (Q1, Q3)
Heart rate (bpm)			Mean (SD)			Mean (SD)	
	T1	6	82.33 (12.86)	80.00 (72.00; 95.50)	7	78.86 (10.56)	75.00 (72.00; 89.00)
	T2	6	86.17 (10.15)	85.50 (76.75; 96.25)	7	88.86 (13.33)	87.00 (83.00; 99.00)
	T3	5	93.40 (13.24)	95.00 (80.50; 105.5)	7	91.57 (8.69)	94.00 (86.00; 96.00)
	T5	4	73.75 (12.01)	73.50 (62.75; 85.00)	5	73.20 (10.38)	72.00 (64.00; 83.00)

Abbreviations: bpm, beats per minute; T, time points (morning baseline (T1), preoperative (T2), intraoperative (T3) and evening (T5)); SD, standard deviation; Q1, Q3, quartile 1, 3.

**TABLE 3:** Heart rate at each surgical stage.

Variables	Timepoint	*n*	Mean (SD)	Median (Q1, Q3)
Heart rate (bpm)				
	Anesthesia	11	89.82 (11.57)	95.00 (78.00; 99.00)
	Incision	11	91.45 (11.33)	93.00 (83.00; 98.00)
	Flap preparation	11	93.45 (9.65)	94.00 (86.00; 98.00)
	Harvesting	10	92.40 (9.43)	90.00 (86.50; 96.25)
	Suturing	11	91.45 (12.02)	93.00 (81.00; 99.00)

Abbreviations: bpm, beats per minute; SD, standard deviation; Q1, Q3, quartile 1, 3.

The average surgical experience in performing soft tissue augmentation using a SCTG was 3.7 years (range: 0–20 years of experience) and the mean surgery duration was 76 min.

### 4.1 Physiological Measurements

#### 4.1.1 Heart Rate (HR)

**FIGURE 3:**
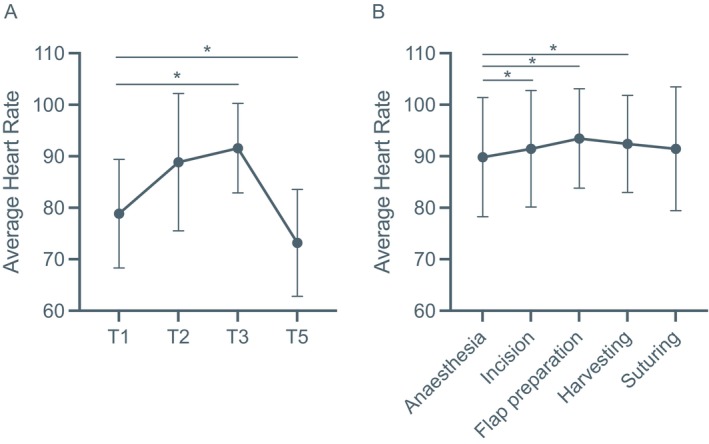
Line plots showing the average heart rates of operating clinicians at the different timepoints (A) and across the different surgical stages (B). Mean heart rate at morning baseline (T1), preoperative (T2), intraoperative (T3) and evening (T5). Differences were tested using a linear mixed‐effect model. * < 0.05 and error bars indicate standard deviation.

A mixed‐effects linear regression model was fitted to examine changes in heart rate (HR) across time (Table 1) and clinician experience (Table 2). A significant overall time effect was detected (*p* < 0.001). Specifically, HR increased at Time 3 compared with baseline (*p* = 0.029) and decreased at Time 5 (*p* = 0.050), indicating a nonlinear pattern over time (Figure 3A). Clinician experience (< 3 years vs. ≥ 3 years) had no significant main effect (*p* = 0.548) and no interaction between experience and time was observed (all *p* > 0.26) (Table 2), indicating similar HR patterns across experience levels. These results indicate that HR varied significantly across time points, showing a transient mid‐procedure increase followed by a return toward baseline.

When HR was analyzed by surgical step (Figure 3B), the lowest were observed during local anesthesia. HR increased during incision and flap preparation and declined during suturing, consistent with physiological activation in response to surgical stimulation (Table 3). Mixed‐effects modeling confirmed a significant main effect of surgical stage, with higher HR during incision (*p* = 0.045), flap preparation (*p* = 0.014), and graft harvesting (*p* = 0.001) compared to baseline, followed by a return toward baseline during suturing (*p* = 0.316) (Figure 3B). Clinician experience (< 3 years vs. ≥ 3 years) did not significantly influence HR changes across surgical stages (all *p* > 0.05).

Overall, heart rate followed a consistent pattern, rising during the most technically demanding phases of surgery and returning to normal as the procedure ended. This pattern suggests short‐term physiological activation related to task demands rather than sustained stress.

#### 4.1.2 Cortisol Levels

**FIGURE 4:**
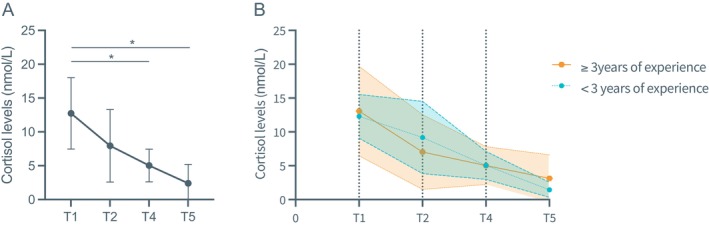
Line plots showing the average heart rates of operating clinicians at the different timepoints (A) and according to clinical experience (B). Cortisol levels at morning baseline (T1), preoperative (T2), postoperative (T4) and evening (T5). Differences were tested using a linear mixed‐effect model. * < 0.05 and error bars and bands indicate standard deviation.

Over the four time points, cortisol levels decreased significantly, especially after the second time point (Figure 4A, Table 4). However, this trend was consistent regardless of the experience of the clinician, meaning experience level did not significantly influence the pattern of change (Table 4B, Table 5). Mean cortisol levels were highest at T1 and decreased significantly at T4 (*p* = 0.002) and T5 (*p* = 0.000) (Figure 4A). These findings indicate a pronounced reduction in circulating cortisol as the procedure advanced, reflecting a gradual attenuation of hypothalamic–pituitary–adrenal (HPA) axis activation and an overall decline in physiological stress.

**TABLE 4:** Cortisol levels at each time point.

Variables	Timepoint	*n*	Mean (SD)	Median (Q1, Q3)
Cortisol (nmol/L)				
	T1	14	12.74 (5.26)	13.50 (9.4; 15.50)
	T2	14	7.95 (5.34)	5.40 (4.10; 13.25)
	T4	14	5.03 (2.41)	4.85 (2.52; 7.30)
	T5	14	2.40 (2.78)	1.00 (1.00; 2.87)

Abbreviations: nmol/L, nanomoles per liter; T, time points (morning baseline (T1), preoperative (T2), postoperative (T4) and evening (T5)); SD, standard deviation; Q1, Q3, quartile 1, 3.

**TABLE 5:** Cortisol levels at each time point stratified by clinical experience.

Variables	Timepoint	*n*	< 3 years of experience	Median (Q1, Q3)	*n*	≥ 3 years of experience	Median (Q1, Q3)
Cortisol (nmol/L)			Mean (SD)			Mean (SD)	
	T1	6	12.30 (3.22)	11.50 (9.4; 15.50)	8	13.08 (6.61)	14.00 (4.15; 17.75)
	T2	6	9.18 (5.34)	9.20 (3.97; 14.25)	8	7.03 (5.53)	4.95 (4.15; 8.17)
	T4	6	5.05 (2.05)	4.58 (3.20; 7.30)	8	5.02 (2.79)	5.00 (2.37; 7.45)
	T5	6	1.45 (1.10)	1.00 (1.00; 1.67)	8	3.12 (3.49)	1.00 (1.00; 6.87)

Abbreviations: nmol/L, nanomoles per liter; T, time points (morning baseline (T1), preoperative (T2), postoperative (T4) and evening (T5)); SD, standard deviation; Q1, Q3, quartile 1, 3.

### 4.2 Psychological Measurements

#### 4.2.1 STAI‐6 Scale

**FIGURE 5:**
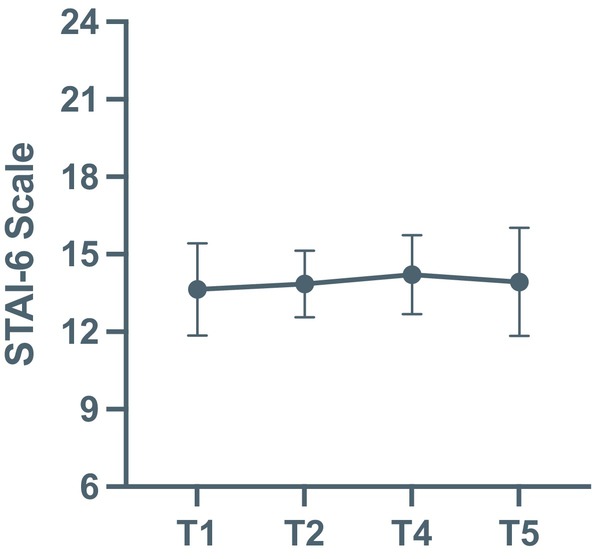
Line plots showing the average STAI‐6 Scale at the different timepoints. At morning baseline (T1), preoperative (T2), postoperative (T4), and evening (T5). Differences were tested using a linear mixed‐effect model.

Self‐reported anxiety measured using the State–Trait Anxiety Inventory (STAI‐6) remained stable across all time points (Figure 5, Table 6). Linear mixed‐effects analysis revealed no significant effect of time or clinician experience (*p* > 0.05), suggesting that anxiety levels did not vary throughout the procedure and followed a similar pattern across experience groups. Although minor differences in baseline anxiety were observed between clinicians, no systematic trend was detected over time. Overall, these findings suggest that clinicians maintained stable levels of state anxiety, consistent with effective emotional regulation despite the transient physiological activation observed during surgery.

**TABLE 6:** STAI‐6 scores each time point.

Variables	Timepoint	*n*	Mean (SD)	Median (Q1, Q3)
STAI‐6				
	T1	14	13.64 (1.78)	14.00 (11.75; 15.00)
	T2	14	13.86 (1.29)	14.00 (13.00; 15.00)
	T4	14	14.21 (1.52)	15.00 (13.00; 15.00)
	T5	14	13.93 (2.09)	15.00 (13.25; 15.00)

Abbreviations: T, time points (morning baseline (T1), preoperative (T2), postoperative (T4) and evening (T5)); SD, standard deviation; STAI‐6, state–trait anxiety inventory; Q1, Q3, quartile 1, 3.

#### 4.2.2 SURG‐TLX Scale

**FIGURE 6:**
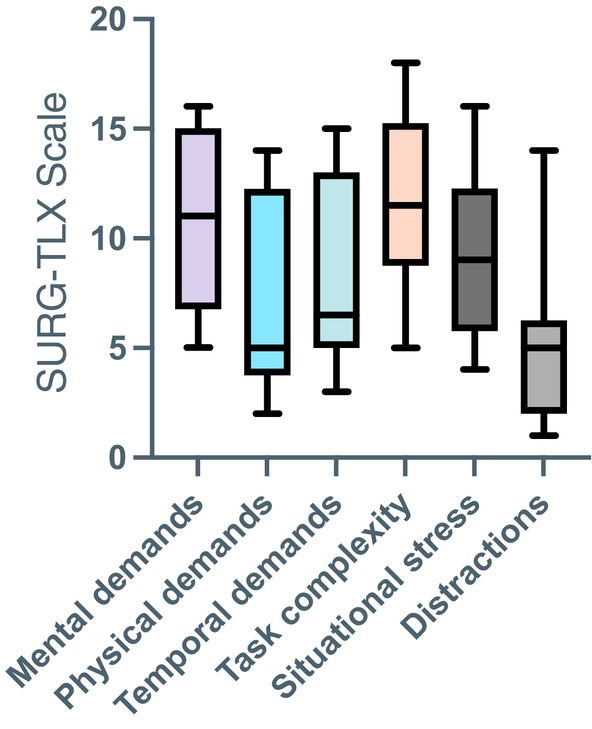
Surgical workload assessment scores using the SURG‐TLX scale.

Results from the SURG‐TLX workload assessment indicated clear differences across different task dimensions (Figure 6). The highest median scores were observed for mental demands (i.e., “How mentally fatiguing was the procedure?”) and task complexity (i.e., “How complex was the procedure?”), followed by situational stress (i.e., “How anxious did you feel while performing the procedure?)” and temporal demand (i.e., “How hurried or rushed was the pace of the procedure?”). In contrast, physical demands (i.e., “How physically fatiguing was the procedure?”) and distractions (i.e., “How distracting was the operating environment?”) received the lowest scores (Figure 6). This pattern suggests that clinicians perceived the procedure as primarily cognitively demanding and technically complex, rather than physically strenuous or heavily influenced by external interruptions. Overall, the findings describe a workload profile dominated by cognitive engagement and focused attention, which is characteristic of fine motor surgical tasks.

## 5 Discussion

This exploratory pilot study provides preliminary descriptive evidence that soft‐tissue augmentation using subepithelial connective tissue grafts (SCTG) for mucosal thickening is associated with measurable physiological stress responses in clinicians. Given the small sample size and exploratory design, these findings should be interpreted cautiously and viewed primarily as hypothesis‐generating.

### 5.1 Physiological Stress Response and Recovery

Heart rate increased during the most technically demanding phases of surgery, likely reflecting the combined cognitive, motor and ergonomic demands of SCTG harvesting, which requires sustained visual attention and precise tissue handling. Similar patterns of transient physiological activation have been reported in laparoscopic and microsurgical settings, although typically at higher absolute stress levels [12, 16]. The subsequent reduction in heart rate during suturing suggests effective autonomic recovery once the most demanding surgical steps were completed.

No significant differences in heart rate responses were observed between less‐ and more‐experienced clinicians. This finding may indicate that the intrinsic technical and cognitive demands of SCTG harvesting elicit comparable physiological engagement across experience levels. Alternatively, the limited sample size, the exploratory nature of the analysis and the arbitrary dichotomization of experience may have reduced sensitivity to detect subtle group differences, precluding firm conclusions regarding experience‐related effects.

Salivary cortisol levels declined progressively throughout the day, consistent with normal diurnal variation. The absence of an intraoperative cortisol peak suggests that SCTG procedures, while inducing short‐term sympathetic activation, do not provoke sustained endocrine stress responses. Together, the heart rate and cortisol findings indicate a well‐regulated physiological response characterized by transient activation and timely recovery, reflecting adaptive stress modulation in response to moderate surgical workload.

### 5.2 Psychological Regulation and Workload Perception

In contrast to physiological measures, self‐reported anxiety assessed using the STAI‐6 remained stable across all time points and did not differ by clinician experience. This dissociation between physiological activation and subjective anxiety has been widely documented in surgical and medical contexts and likely reflects effective emotional regulation and cognitive control among trained clinicians [17, 18]. Despite measurable autonomic responses, participants perceived themselves as calm and focused throughout the procedure.

Surgical workload assessment using the SURG‐TLX revealed that mental demand and task complexity were perceived as the most challenging dimensions, followed by situational stress and time pressure. Physical demands and external distractions were minimal. These findings emphasize the cognitive rather than emotional or physical burden of SCTG surgery and support its classification as a mentally intensive fine‐motor surgical task [13, 19, 20]. Similar observations have been reported in microsurgical and laparoscopic fields [9, 10], where cognitive load consistently represents the dominant component of surgical workload.

### 5.3 Integration and Implications

Taken together, the findings suggest that SCTG procedures elicit a moderate, adaptive stress response, characterized by transient physiological activation, normal endocrine regulation and stable perceived anxiety. While these responses may be intuitive to experienced clinicians, the present study provides empirical documentation of stress dynamics within the context of mucogingival surgery.

Although stress‐management and ergonomic awareness are widely promoted in surgical education, this study did not evaluate specific interventions or define clinically relevant stress thresholds. As such, any implications for training or workflow optimization should be considered conceptual rather than evidence based. Nevertheless, the observed adaptive stress profile aligns with the inverted‐U model of arousal, in which moderate physiological activation supports sustained attention and technical performance. The comparable responses across experience levels suggest that procedural complexity itself, rather than clinician expertise alone, may be a key determinant of physiological load during SCTG surgery.

From a practical perspective, these findings underscore that stress is an inherent and regulated component of mucogingival surgery, particularly in procedures requiring prolonged precision and focused attention. Future studies should examine how psychophysiological stress responses relate to objective performance metrics, ergonomic posture, learning curves and clinical outcomes (e.g., optimal or suboptimal outcomes) and whether targeted interventions can enhance long‐term performance and clinician resilience in dental microsurgery.

Key limitations include the small sample size, heterogeneity in clinician experience and the absence of objective performance or patient outcome measures. In addition, given the exploratory design and limited sample size, no adjustment was performed for potential confounders such as baseline stress traits, workload or other factors that could influence heart rate or cortisol levels. These limitations restrict causal interpretation and underscore the exploratory, hypothesis‐generating nature of the study.

## 6 Conclusion

In this exploratory pilot study, SCTG procedures were associated with transient physiological stress activation without corresponding increases in perceived anxiety. These findings suggest adaptive stress regulation during mucogingival surgery. Larger, adequately powered studies are needed to clarify the relationship between intraoperative stress, technical performance, ergonomics and clinical outcomes and to determine whether targeted interventions can meaningfully improve clinician well‐being or procedural quality.

## Disclosure

The authors have nothing to report.

## Conflicts of Interest

The authors declare no conflicts of interest.

## Data Availability

The data that support the findings of this study are available from the corresponding author upon reasonable request.
